# AtSPX1 affects the AtPHR1–DNA-binding equilibrium by binding monomeric AtPHR1 in solution

**DOI:** 10.1042/BCJ20170522

**Published:** 2017-10-23

**Authors:** Wanjun Qi, Iain W. Manfield, Stephen P. Muench, Alison Baker

**Affiliations:** 1 School of Molecular and Cellular Biology, Centre for Plant Sciences and Astbury Centre for Structural Molecular Biology, Faculty of Biological Sciences, University of Leeds, Leeds LS2 9JT, U.K.; 2 Astbury Centre for Structural Molecular Biology, Faculty of Biological Sciences, University of Leeds, Leeds LS2 9JT, U.K.; 3 School of Biomedical Sciences, Faculty of Biological Sciences, University of Leeds, Leeds LS2 9JT, U.K.

**Keywords:** *Arabidopsis thaliana*, eukaryotic gene expression, myb transcription factor, phosphate, surface plasmon resonance

## Abstract

Phosphorus is an essential macronutrient for plant growth and is deficient in ∼50% of agricultural soils. The transcription factor phosphate starvation response 1 (PHR1) plays a central role in regulating the expression of a subset of phosphate starvation-induced (PSI) genes through binding to a *cis*-acting DNA element termed P1BS (PHR1-binding sequences). In *Arabidopsis* and rice, activity of AtPHR1/OsPHR2 is regulated in part by their downstream target SPX (Syg1, Pho81, Xpr1) proteins through protein–protein interaction. Here, we provide kinetic and affinity data for interaction between AtPHR1 and P1BS sites. Using surface plasmon resonance, a tandem P1BS sequence showed ∼50-fold higher affinity for MBPAtdPHR1 (a fusion protein comprising the DNA-binding domain and coiled-coil domain of AtPHR1 fused to maltose-binding protein) than a single site. The affinity difference was largely reflected in a much slower dissociation rate from the 2× P1BS-binding site, suggesting an important role for protein co-operativity. Injection of AtSPX1 in the presence of phosphate or inositol hexakisphosphate (InsP6) failed to alter the MBPAtdPHR1-P1BS dissociation rate, while pre-mixing of these two proteins in the presence of either 5 mM Pi or 500 µM InsP6 resulted in a much lower DNA-binding signal from MBPAtdPHR1. These data suggest that, in the Pi-restored condition, AtSPX1 can bind to monomeric AtPHR1 in solution and therefore regulate PSI gene expression by tuning the AtPHR1–DNA-binding equilibrium. This Pi-dependent regulation of AtPHR1–DNA-binding equilibrium also generates a negative feedback loop on the expression of AtSPX1 itself, providing a tight control of PSI gene expression.

## Introduction

Phosphorus (P) is well recognized for its importance in multiple biological processes; however, its bioavailable form, inorganic phosphate (Pi), is environmentally limited. Binding to metals and absorption on the surface of soil particles often renders the free Pi concentration in soil 1000 times lower than that in plant intracellular compartments [1]. To alleviate the economic [2] and environmental [3] stresses of Pi fertilizer application and to increase the Pi use efficiency, the natural strategies plants use to cope with Pi deprivation have been studied intensively. During Pi starvation, enhanced Pi acquisition is often achieved by alterations in the root system architecture [4,5], followed by the increased expression of high-affinity Pi transporters from the phosphate transporter 1 (PHT1) family [6–8]. The plant intracellular Pi level is also balanced by Pi efflux via the PHO1 family members [9,10] as well as vacuolar Pi storage via the tonoplast transporter vacuolar phosphate transporter 1 [11]. Post-transcriptional regulation by non-coding RNAs and post-translational regulation by protein trafficking and degradation are also identified to play important roles in plant Pi regulation [12–15].

Expression of a significant subset of Pi-related genes, including *PHT1*s, *PHO1;H1* and *SPX* (Syg1, Pho81, Xpr1) family members, has been found to be regulated by an Myb-CC transcription factor, phosphate starvation response 1 (PHR1) through interaction with the P1BS in the gene promoter regions [9,16–18]. It has been established that this regulation function of PHR1 is under the control of an upstream small ubiquitin-like modifier (SUMO) E3 ligase, SIZ1 [19]. Recent studies in *Arabidopsis* and rice demonstrated that the activity of PHR1 is negatively regulated by its downstream targets, the SPX family members, in a Pi-dependent way [20–22].

The SPX family members belong to a subfamily of plant SPX domain-containing proteins, with those containing this well-conserved N-terminal hydrophilic SPX domain being first identified in the yeast *Saccharomyces cerevisiae* (reviewed in ref. [23]). The connection between SPX domain-containing proteins and Pi homeostasis was quickly established after 8 out of 10 yeast SPX domain-containing proteins were identified to participate in different aspects of Pi regulation [24–29]. In plants, the SPX domain-containing proteins are classified into four subfamilies depending on the nature of the C-terminal extra domains [23]. Although members from all four subfamilies have been found to contribute to plant Pi regulation [13,18,30–34], the exact function of this well-conserved SPX domain was unclear due to a lack of structural information. However, recently the crystal structure of the SPX domain showed that it can physically interact with Pi and therefore act as an intracellular Pi sensor [35]. The same study also found that ligand binding triggers the conformational changes of SPX domains, which might involve the intrinsically disordered N-terminal α-1 helix [35]. Furthermore, as the SPX domain binds to inositol polyphosphate (InsPs) with a much higher affinity than to Pi, it is proposed that InsPs rather than Pi is the physiologically relevant signaling molecule [35].

However, in spite of these latest advances, the mechanism of AtSPX1–AtPHR1 interaction and its effect on the regulation of PSI (phosphate starvation-induced) gene expression and intracellular Pi level are still unclear, due to lack of quantitative data on kinetics, affinity and order of binding between the different components of the system. The present study in *Arabidopsis* using surface plasmon resonance (SPR) reports quantitative data on AtPHR1–DNA interaction and the effect of AtSPX1–AtPHR1 interaction on DNA binding. The results support a model where protein co-operativity and the P-sensing protein AtSPX1 influence the AtPHR1–DNA-binding equilibrium to regulate the downstream PSI gene expression and subsequently keep the intracellular Pi level in balance.

## Experimental procedures

### Plasmid construction

pGEX-2T-AtSPX1 was generated for expression of GST–AtSPX1, by subcloning cDNA encoding full-length AtSPX1 (obtained from the Arabidopsis Biological Resource Centre, At5G20150.1) into pGEX-2T GST Expression Vector (GE Healthcare) via the *Bam*HI and *Eco*RI sites. For expression of MBP–AtdPHR1, the gene encoding a truncated version of AtdPHR1 (amino acids 208–362) was amplified by polymerase chain reaction (PCR) using full-length AtPHR1 cDNA (obtained from the Arabidopsis Biological Resource Centre, AT4G28610.1) as a template and KOD hot start polymerase (Novagen). The forward primer used was 5′-GGATCCGAATTGCGACCTGTTAGCACAAC-3′, containing a *Bam*HI restriction site. The reverse primer used was 5′-AAGCTTCACCCTTTGGTAAGACCAGAGTTTTG-3′, containing a *Hin*dIII restriction site. The PCR product of AtdPHR1 was subcloned into pMAL-c2x expression vector (New England BioLabs, Inc.). The pGEX-4T GST vector was used for expression of GST protein. These constructs are directly equivalent to those reported in ref. [20].

### Protein expression and purification

Expression of GST–AtSPX1 and GST was induced in *Escherichia coli* BL21 (DE3) cells with 0.1 mM IPTG at OD_600_ ∼0.5. Induced cells containing GST–AtSPX1 or GST were grown at 18°C overnight or at 37°C for 2 h. Cells were harvested and lysed using a cell disrupter (Constant Systems Ltd) at 30 kpsi twice. GST–AtSPX1 and GST proteins were affinity-purified at 4°C using Glutathione Sepharose 4 Fast Flow resin (GE Healthcare) according to the manufacturer's instructions. Purified GST–AtSPX1 and GST were flash-frozen and stored at −80°C.

Expression of MBP–AtdPHR1 was conducted in an *E. coli* BL21-gold (DE3) cell strain according to the pMAL protein fusion and purification system instruction manual (New England BioLabs, Inc.). Cells were harvested and lysed as above. MBP–AtdPHR1 protein was affinity-purified at 4°C using Amylose Resin (New England BioLabs, Inc.) according to the manufacturer's instructions. The affinity-purified protein was dialyzed into 10 mM HEPES (pH 7.5), 50 mM NaCl and further purified via ion exchange chromatography (IEX) on a HiTrap Q-HP 1 ml column (GE Healthcare) with a gradient from 50 mM to 1 M NaCl in 10 mM HEPES (pH 7.5) using an ÄKTA Explorer (GE Healthcare). Purified MBP–AtdPHR1 was flash-frozen and stored at −80°C.

Size-exclusion chromatography (SEC) on IEX-purified MBP–AtdPHR1 was performed on a Superdex 200 10/300 GL column (GE Healthcare) in SPR buffer [10 mM HEPES (pH 7.5) and 200 mM NaCl]. The void volume of the column was determined using Blue Dextran 2000, and the partition coefficient *K*_av_ for individual proteins was calculated as follows: *K*_av_  =  (*V*_r_ − *V*_o_)/(*V*_c_ − *V*_o_) according to the Superdex 200 HR 10/30 instruction (GE Healthcare). A calibration curve of protein molecular weight was generated by plotting log *M*_w_ against *K*_av_ values for standard proteins ovalbumin (44 kDa), conalbumin (75 kDa), ferritin (440 kDa), aldolase (158 kDa) and thyroglobulin (669 kDa).

### Sodium dodecyl sulfate–polyacrylamide gel electrophoresis and immunoblotting

Protein purification fractions and pull-down fractions were analyzed using sodium dodecyl sulfate–polyacrylamide gel electrophoresis (SDS–PAGE). Samples were suspended in SDS loading buffer, incubated at 95°C for 5 min and run on 12% polyacrylamide gels. Protein bands were then fixed with 25% isopropanol, 10% acetic acid and stained with 0.025% Coomassie Blue R-250. Background staining was removed with 10% acetic acid before imaging.

For immunoblotting, proteins were transferred to an Amersham Nitrocellulose Western blotting membrane (GE Healthcare) after SDS–PAGE, using a Trans-Blot Turbo Transfer System (Bio-Rad) for 20 min at 25 V. Membranes were blocked in 3% (w/v) bovine serum albumin (BSA) in TBS-T [Tris-buffered saline with 0.1% (v/v) Tween] for 2 h at room temperature. The membranes were probed with polyclonal rabbit anti-glutathione-*S*-transferase (GST) antibody (1 : 2000) (Sigma–Aldrich, G7781) to detect GST-tagged proteins. Horseradish peroxidase-conjugated secondary goat anti-rabbit antibody (Jackson ImmunoResearch Lab, 111-035-003) was then used at a concentration of 1 : 20 000. Proteins were visualized by chemiluminescence after treatment with SuperSignal West Pico Chemiluminescent Substrate (Thermo Fisher Scientific).

### Pull-down assay

To qualitatively detect the interaction between GST–AtSPX1 and MBP–AtdPHR1, MBP–AtdPHR1 was expressed and purified as described above except that the lysis buffer was either with Pi [PBS (pH 7.4), 200 mM NaCl, 1 mM EDTA and 1 mM DTT] or without Pi [20 mM Tris (pH7.5), 200 mM NaCl, 1 mM EDTA and 1 mM DTT] in the presence of cOmplete EDTA-free Protease Inhibitor Cocktail (Roche). MBP-tagged constructs in the cell lysates were immobilized to amylose affinity matrix for 2 h at 4°C. MBP–AtdPHR1-bound resin was washed with 8CV (column volume) of the same lysis buffers and incubated with affinity-purified GST–AtSPX1 for 2 h at 4°C. Any unbound proteins were removed by washing with 6CV the same lysis buffers and bound proteins were eluted with 3CV same lysis buffers containing 10 mM maltose. In the control pull-down experiment, GST–AtSPX1 was replaced with GST protein. Samples of unbound flow-through and elution fractions were suspended in SDS gel-loading buffer, analyzed on SDS–PAGE gels and blotted with anti-GST antibody.

### DNA probe preparation

P1BS probes containing 1× P1BS motif and 2× P1BS motifs were taken from the promoter regions of AtSPX3 (−192 to −139 bp) and AtSPX1 (−136 to −83 bp), respectively. Single forward strand DNA molecules were synthesized and biotinylated (Sigma–Aldrich) on the 5′-end: 5′-ACACTTCGTCACGCTAAAGCTAAGCATATCCGCTTTCATATTCCTTTACACAAC-3′ for the 1× P1BS probe and 5′-CAGAGAAAAAAGGATATTCTAATTAGAAACCTTAAGAATATTCTTTTTAATCCC-3′ for the 2× P1BS probe (P1BS motifs are underlined). Reverse complementary strands were synthesized without biotinylation. The control DNA probe with met-box sequences (5′-CCGGCAGGAGACGTCTAGACGTCTCCGGCAGG-3′) does not contain P1BS motifs.

DNA oligos were dissolved to 100 µM in TE buffer according to the manufacturer's instructions (Sigma–Aldrich). Single-strand biotin-labeled and non-biotin-labeled DNA oligos were diluted in TM buffer [10 mM Tris (pH 7.5) and 10 mM MgCl_2_] to a final concentration of 10 and 11 µM, respectively, annealed at 95°C for 1 min and cooled slowly to room temperature. Annealed P1BS DNA probes were kept at −20°C before use.

### SPR studies

SPR experiments were performed on a Biacore 3000 instrument (GE Healthcare). Biotinylated DNA probes at 10 nM were immobilized on Streptavidin (SA) sensor chips (GE Healthcare) with contact times of 6–8 min and a flow rate of 5 µl/min, to give ∼500 RU of immobilized DNA. The reference flow cell was underivatized. All ligand immobilization was done in HEPES-buffered saline consisting of 10 mM HEPES (pH 7.5), 200 mM NaCl and 0.01% Surfactant P-20. Analyte measurements were carried out at 25°C and a flow rate of 40 µl/min, using the same buffer with or without additional Pi/InsP6 (inositol hexakisphosphate). For MBP–AtdPHR1–DNA-binding assays, 120 µl of IEX-purified MBP–AtdPHR1 was injected in a two-fold ascending concentration, commencing at 0.3125 nM and ending at 80 nM. For sequential injection experiments, the ‘Coinject’ command (Biacore 3000 Instrument Handbook, GE Healthcare) was used to inject 100 µl of the first component immediately followed by 120 µl of the second component. For pre-mixing injection experiments, 120 µl of premixed proteins were injected over the chip surface using the ‘Inject’ command.

The chip surface was regenerated between protein injections with a 40 µl 0.05% SDS injection.

The binding data were analyzed using the BIAevaluation 3.1 software (GE Healthcare). To derive affinity and kinetic parameters, data from binding assays at 2.5 nM were used where significant binding signal was seen on a 1× P1BS site and giving acceptable fits to a 1 : 1 model.

IP6 was phytic acid Na salt hydrate (Sigma P8810).

## Results

### MBPdAtPHR1 binds with higher affinity to tandem P1BS than to single P1BS

**Figure 1.**
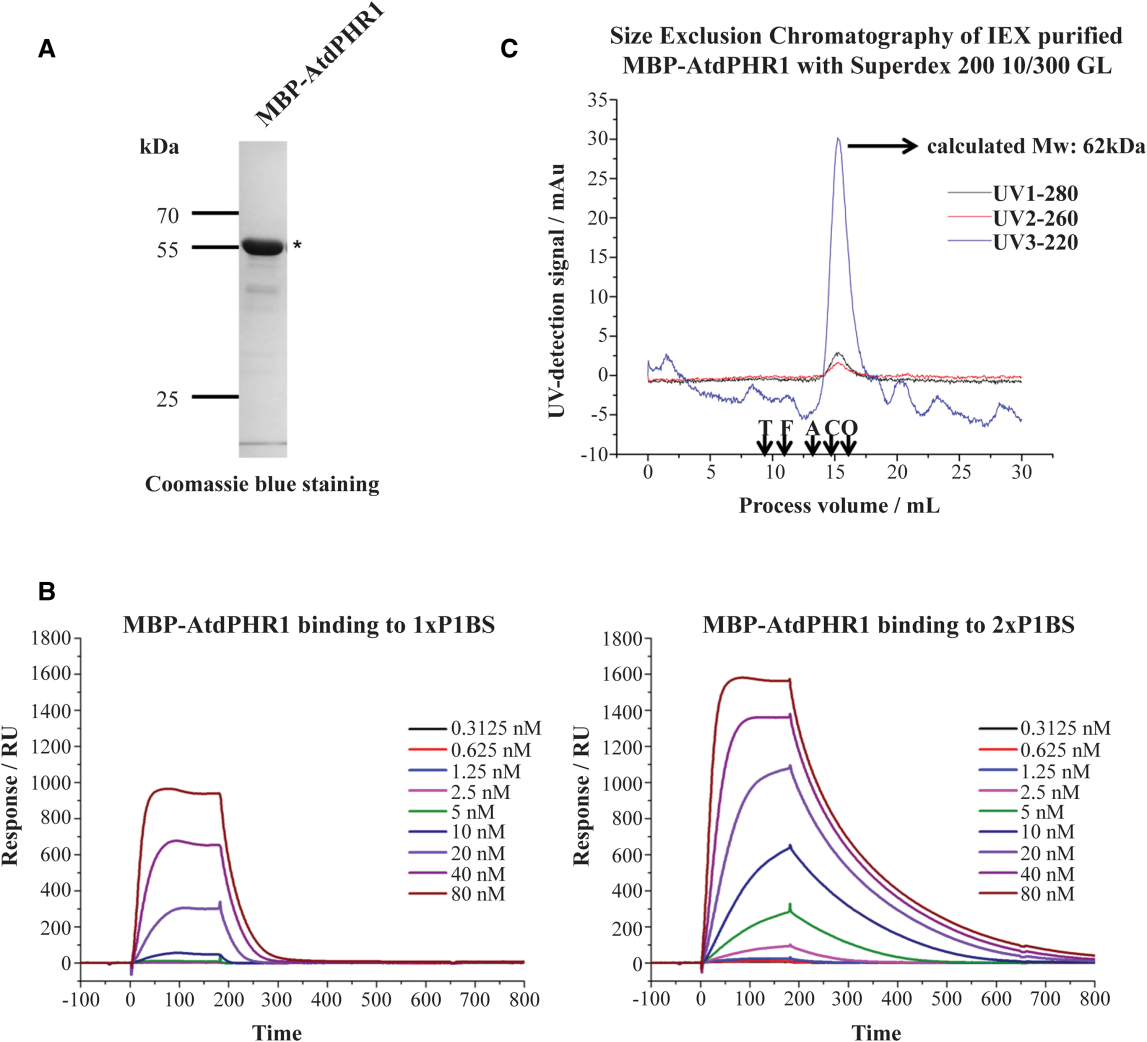
MBP–AtdPHR1 is a monomeric protein which binds with higher affinity to tandem P1BS motifs compared with a single P1BS motif. (**A**) IEX purification of affinity-purified MBP–AtdPHR1. MBP–AtdPHR1 is indicated by *. (**B**) SPR plots of MBP–AtdPHR1 interaction with immobilized DNA probes containing 1× P1BS (left panel) and 2× P1BS (right panel). Concentrations of MBP–AtdPHR1 are in a two-fold ascending order, commencing at 0.3125 nM and ending at 80 nM. (**C**) MBP–AtdPHR1 is a monomeric protein at 5 µM concentration with a single peak at 62 kDa on SEC. Elution volumes of standard proteins are indicated by ↓ on the *X*-axis. Molecular mass of standard proteins: Thyroglobulin (T): 669 kDa; Ferritin (F): 440 kDa; Aldolase (A): 158 kDa; Conalbumin (C): 75 kDa; Ovalbumin (O): 44 kDa.

To understand the binding of the transcription factor AtPHR1 (Supplementary Figure S1A) to its target DNA molecules more precisely, we measured the interaction of AtPHR1 and its DNA target P1BS using SPR. N-terminal MBP-tagged recombinant AtdPHR1 (aa 280–362), which lacks the transcription activation domains (Supplementary Figure S1B), was purified using affinity media followed by IEX to remove the associated DNA from the *E. coli* expression strain host (Figure 1A). IEX-purified MBP–AtdPHR1 was tested for its binding capacity to P1BS motifs using two-fold ascending concentrations, ranging from 0.31 to 80 nM. The binding profiles showed that the binding signal to P1BS flow-cells gets stronger with increasing MBP–AtdPHR1 concentration (Figure 1B); however, no binding was seen to the negative control site without P1BS (data not shown). Analysis of sensorgram data obtained at low PHR1 concentrations showed that the affinity for 1× P1BS was 50-fold lower than that for 2× P1BS (Table 1). SPR allows the dissection of affinity into association and dissociation rates, and this showed a small difference in the association rate (1.7× faster for the 2× P1BS) but a 30 times slower dissociation rate (Table 1). This suggests that protein co-operativity and possible dimerization of MBP–AtdPHR1 are occurring during its interaction with P1BS.

**Table 1:** Affinity and kinetic parameters of MBP–AtdPHR1 binding to P1BS sites Data derived using a 1 : 1 binding model with data from a binding assay at 2.5 nM (*n* = 3).

	1× P1BS	2× P1BS	Ratio 1× P1BS : 2× P1BS
*K*_D_ (nM)	707 ± 340	14 ± 2.4	∼50
*k*_a_ (M^−1^ s^−1^)	2.1 × 10^4^	3.5 × 10^4^	∼0.6
*k*_d_ (s^−1^)	0.015	0.00049	∼30

### MBPdPHR1 is monomeric in solution

Dimerization of AtPHR1 has been previously observed during DNA binding [16]; however, it is not clear if PHR1 dimerizes upon binding or exists as a dimer before binding. Therefore, the protein oligomeric state of MBP–AtdPHR1 in solution was examined using SEC. A single 280 nm absorbance peak was observed from SEC when 5 µM of IEX-purified MBP–AtdPHR1 was applied (Figure 1C). The calculated molecular mass of this MBP–AtdPHR1 peak is 62 kDa, in good agreement with the predicted molecular mass of 60 651, corresponding to a monomeric protein state. This result is also corroborated by native mass spectrometry which detected a predominantly monomeric state with <20% dimeric (data not shown). Given that the range of concentrations of MBP–AtdPHR1 used in SPR is 60–1000 times lower than that in SEC, the results show that the transcription factor MBP–AtdPHR1 is monomeric in solution.

### AtSPX1 binds MBPdPHR1 in a Pi-dependent manner

We next examined the ability of AtSPX1 to interact with AtPHR1 and to interfere with the AtPHR1–DNA interaction. Obtaining soluble AtSPX1 was very challenging, and multiple constructs were screened under a wide range of conditions. The N-terminal GST-tagged full-length AtSPX1 (GST–AtSPX1; Supplementary Figure S1C) had the best solubility and recovery after purification, and was therefore used for all the subsequent experiments. Affinity purification of GST–AtSPX1 using glutathione resin showed multiple smaller protein bands (Supplementary Figure S2A). Attempts to remove these by a subsequent IEX purification step resulted in significant loss of the GST–AtSPX1 fusion protein. Alternative buffer conditions were tested using a light-scattering aggregation assay with an Optim 1000 instrument (Unchained Labs), but little improvement in the stability or purity of GST–AtSPX1 could be obtained (data not shown). Nevertheless, western blotting of the affinity-purified GST–AtSPX1 demonstrated that most of the faster migrating proteins are either degradation products of GST–AtSPX1 or free GST tags (Supplementary Figure S2A). Therefore, free GST protein was used as a negative control in a qualitative pull-down assay to test the functionality of the GST–SPX1 and verify the previously observed Pi-dependent interaction between recombinant AtSPX1 and MBP–AtdPHR1 [20] (Supplementary Figure S2B) Quantification showed that 1.5- and 3.5-fold more GST–SPX1 is present in elution fractions E1 and E2 in the presence of phosphate compared with the absence of phosphate (Supplementary Figure S2B, lower panel).

### AtSPX1 blocks MBP–AtdPHR1 binding to DNA but does not displace bound protein from its target site

**Figure 2.**
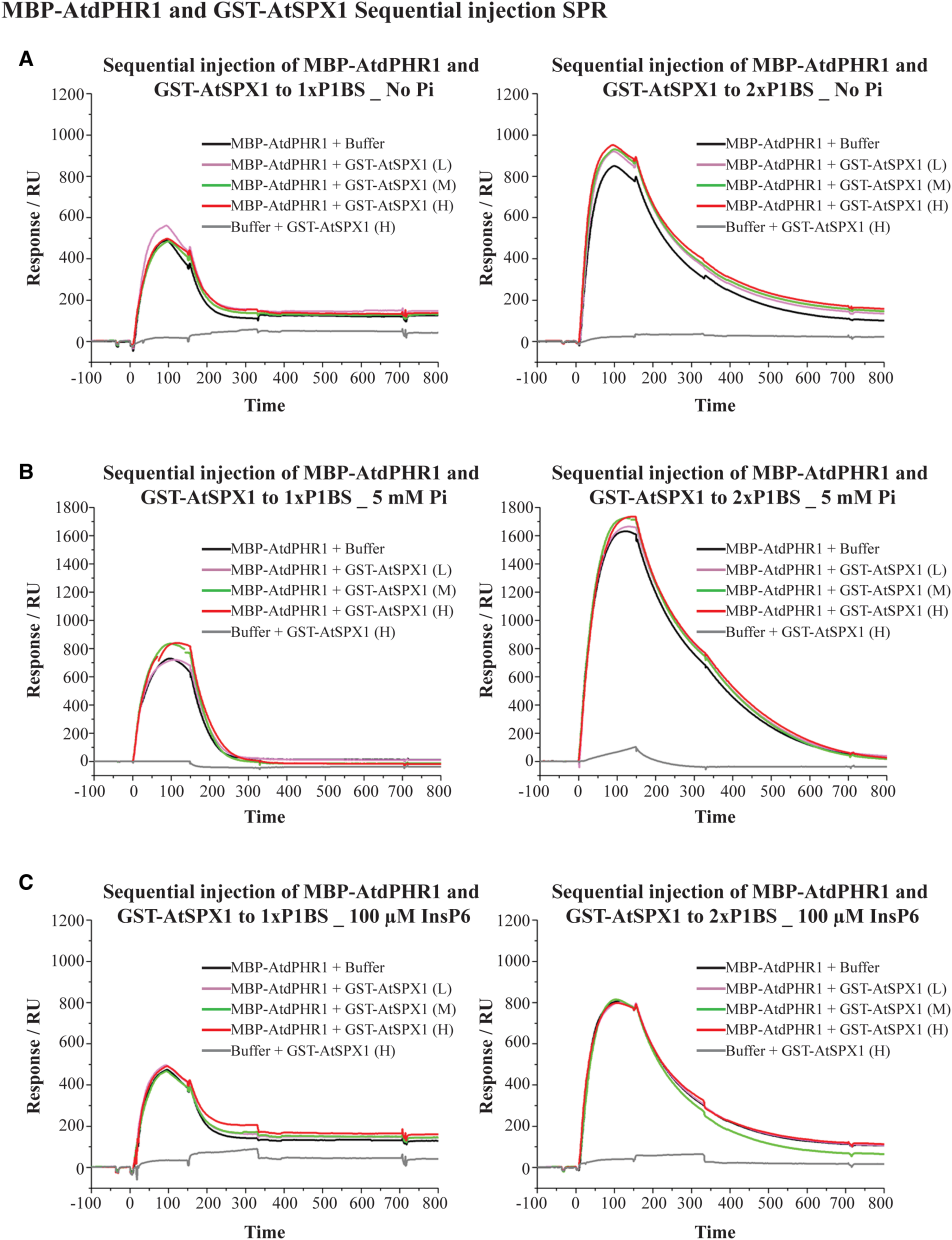
GST–AtSPX1 cannot disrupt preformed MBP–AtdPHR1–P1BS complexes in the presence of up to 5 mM Pi or 100 µM InsP6. SPR plots of sequential injection of MBP–AtdPHR1 and GST–AtSPX1 to immobilized DNA probes containing 1× P1BS (left panels) and 2× P1BS (right panels) in the presence of no Pi (**A**), 5 mM Pi (**B**) and 100 µM InsP6 (**C**). Each experiment contained five injections, commencing with 80 nM MBP–AtdPHR1 + buffer, followed by 80 nM MBP–AtdPHR1 + low concentration (∼40 nM) of GST–AtSPX1, 80 nM MBP–AtdPHR1 + medium concentration (∼200 nM) of GST–AtSPX1, 80 nM MBP–AtdPHR1 + high concentration (∼1000 nM) of GST–AtSPX1 and buffer + high concentration (∼1000 nM) of GST–AtSPX1.

Having shown the interaction between recombinant GST–AtSPX1 and MBP–AtdPHR1 proteins, the interaction mechanism was investigated. Two different binding models of AtSPX1 and AtPHR1 with or without 5 mM Pi were tested using SPR. In the first binding experiment, we examined whether AtSPX1 is able to displace AtPHR1 from its target DNA molecules. MBP–AtdPHR1, at 80 nM, was applied to the P1BS probes, immediately followed by a second injection containing either buffer or varying concentrations of GST–AtSPX1. The SPR result showed that including different concentrations of GST–AtSPX1 with or without 5 mM Pi does not alter either the equilibrium-binding signal or the dissociation rate of the AtdPHR1–DNA interaction. This is true for both the 1× and 2× P1BS-binding site probes (Figure 2A,B). Different concentrations of GST–AtSPX1 showed a similar effect to buffer alone, indicating that GST–AtSPX1 cannot remove MBP–AtdPHR1 from the associated P1BS motifs. A negative control sequential injection containing SPR buffer and the highest concentration of GST–AtSPX1 only displayed a low signal that is close to baseline (Figure 2A,B), showing that the GST–AtSPX1 protein does not bind to P1BS motifs and the low signal is probably caused by non-specific protein binding to the chip surface.

**Figure 3.**
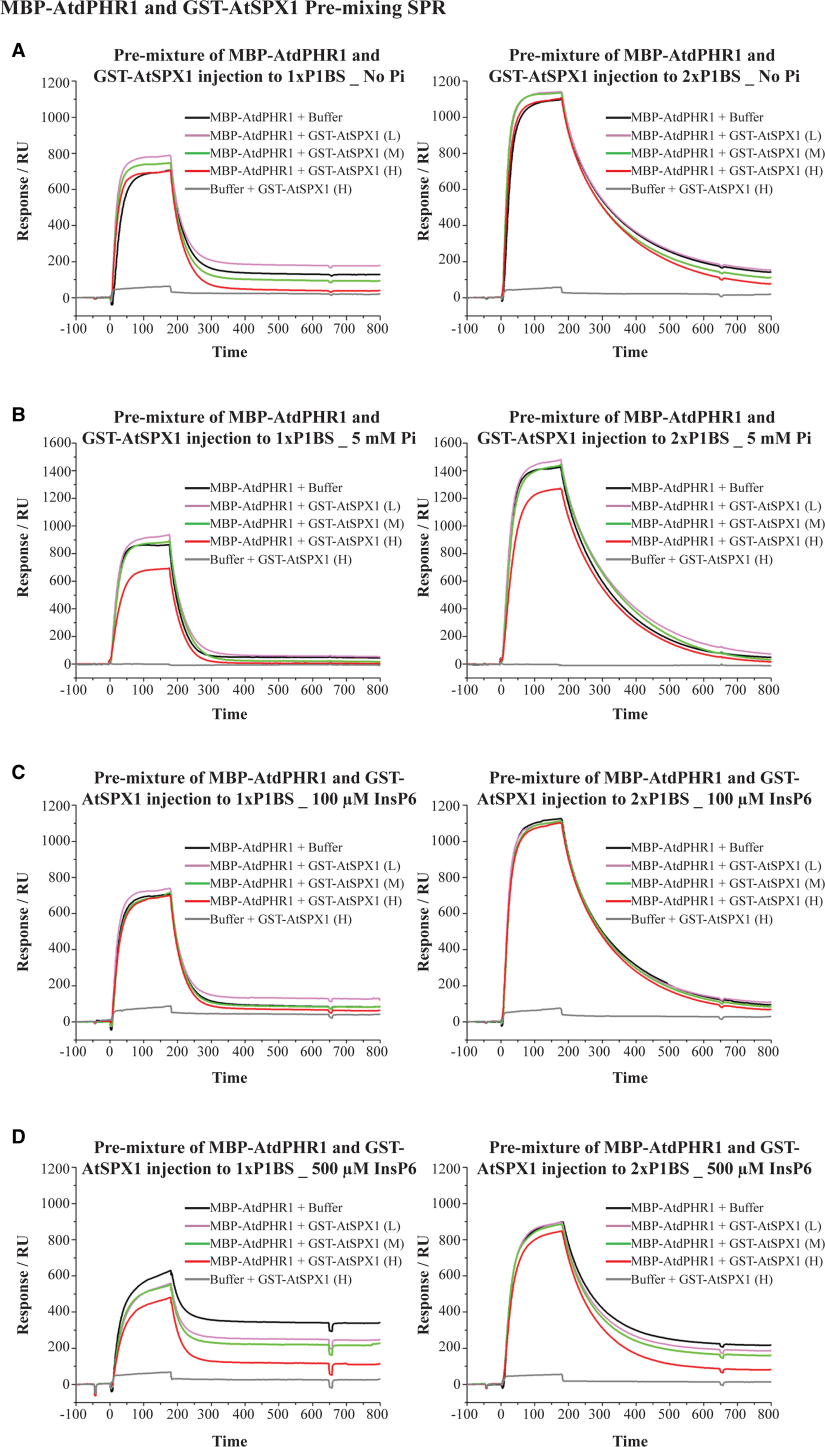
GST–AtSPX1 can prevent free MBP–AtdPHR1 from binding to P1BS motifs in the presence of either 5 mM Pi or 500 µM InsP6. SPR plots of premixed MBP–AtdPHR1 and GST-AtSPX1 samples injected over immobilized DNA probes containing 1× P1BS (left panels) and 2× P1BS (right panels) in the presence of no phosphorus (**A**), 5 mM Pi (**B**), 100 µM InsP6 (**C**) and 500 µM InsP6 (**D**). Each experiment contained five mixtures: 80 nM MBP–AtdPHR1 + buffer, 80 nM MBP–AtdPHR1 + low concentration (∼40 nM) of GST-AtSPX1, 80 nM MBP–AtdPHR1 + medium concentration (∼200 nM) of GST-AtSPX1, 80 nM MBP–AtdPHR1 + high concentration (∼1000 nM) of GST-AtSPX1 and buffer + high concentration (∼1000 nM) of GST-AtSPX1. Mixed samples were incubated on ice for 30 min before being injected onto the immobilized DNA probes.

In the second binding experiment, we tested if the formation of the AtSPX1–AtPHR1 complex could prevent AtPHR1 from binding to P1BS motifs. MBP–AtdPHR1 (80 nM) was pre-mixed with either SPR buffer or varying concentrations of GST–AtSPX1 and incubated to allow any protein complexes to form, before the protein mixture was injected onto the chip surface with the bound P1BS probes. Consistent with previous results, there is a higher equilibrium signal and a slower dissociation rate for the double P1BS site compared with the single site (Figures 1B and 3A,B). Compared with the DNA-binding signal generated by MBP–AtdPHR1 alone, a slightly higher signal was seen when MBP–AtdPHR1 was pre-mixed with low and medium concentrations of GST–AtSPX1 without Pi. This is probably caused by non-specific protein binding which is also seen in the negative control of pre-mixed SPR buffer and GST–AtSPX1 (Figure 3A). However, the equilibrium-binding signal of MBP–AtdPHR1 was decreased by pre-mixing MBP–AtdPHR1 with a high concentration of GST–AtSPX1 in the presence of 5 mM Pi (Figure 3B). No significant change of dissociation rate of MBP–AtdPHR1 and P1BS motifs was observed in this experiment, suggesting that GST–SPX1 can only interact with free MBP–AtdPHR1 and that GST–SPX1 and P1BS motifs may share the same or very close binding sites on MBP–AtdPHR1. A higher proportion of binding signal loss is also seen from the single P1BS probe (Figure 3B), indicating that multiple P1BS motifs could stabilize the interaction between MBP–AtdPHR1 and DNA molecules.

In the course of this work, the SPX domain was demonstrated to bind InsP6 with a much higher affinity than Pi [35]. Therefore, we also examined the effect of InsP6 on the AtSPX1–MBP–AtdPHR1 interaction using the same settings in SPR. In the presence of 100 µM InsP6, GST–AtSPX1 cannot remove MBP–AtdPHR1 from associated DNA or lower the equilibrium MBP–AtdPHR1-binding signal (Figures 2C and 3C). However, when the concentration of InsP6 was increased to 500 µM, pre-mixing with a high concentration of GST–AtSPX1 was able to decrease the equilibrium-binding signal of MBP–AtdPHR1, with a greater signal reduction from the single P1BS motif (Figure 3D).

## Discussion

When facing low environmental Pi stress, plants adopt multiple strategies such as root architecture reprogramming, gene expression profile changes and protein post-translational regulation to increase the absorption and remobilization of this essential element in order to maintain internal Pi homeostasis. Multiple transcription factors have been shown to play critical roles in these processes [36]. In *Arabidopsis*, it has been established that a subset of PSI genes, including high-affinity Pi transporter AtPHT1 family members, are regulated by the transcription factor AtPHR1 through interaction with the P1BS motifs in the promoter regions of the target genes [16]. In this report, we provide novel quantitative and mechanistic insights into the SPX1–PHR1–P1BS interaction.

**Figure 4.**
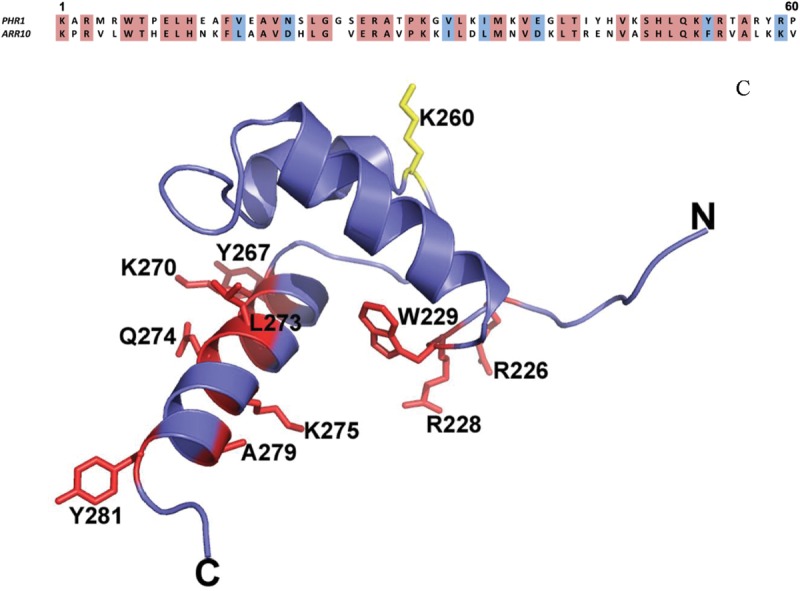
Model of PHR1 DNA-binding domain generated by Phyre II, using 1IRZ solution structure of ARR10B as the template. Alignment of PHR1 and ARR10-B–DNA-binding domains which share 54% identity and 67% similarity in this region. Helices 2 and 3 are a variant of a helix-turn-helix motif; α3 is the presumed recognition helix. Residues colored in red are those amino acids in PHR1 that occupy the same position in the alignment as those that bind DNA in the ARR10-B structure. Yellow indicates the lysine that is modified by SUMOylation.

MBP–AtdPHR1 is predominantly monomeric in solution, but binding to the 2× P1BS motif gave a higher signal arising from the greater affinity and slower dissociation from the 2× P1BS oligonucleotide, pointing to binding interactions between MBP–AtdPHR1 molecules stabilizing the interaction with the DNA. Rubio et al*.* [16] identified the PHR1 protein as a Myb-CC family member possessing both a Myb–DNA-binding domain and a predicted coiled-coil domain (aa 310–355) [16], although the DNA-binding domain is most closely related to the SHAQKYF subclass found in ARR10B [37] to which it shares 54% identity and 67% similarity. Modeling the AtPHR1domain on the structure of ARR10B (pdb 1IRZ) shows that the residues that contact DNA in the ARR10B structure are conserved (Figure 4). A single DNA-binding motif from ARR10B is competent to bind DNA [37]; however, electrophoresis mobility shift assay (EMSA) experiments with a longer PHR1 construct (NΔ207) demonstrated that PHR1 binds the P1BS as a dimer and that the intact coiled-coil domain is important for the high-affinity P1BS motif binding [16]. The P1BS site is an (im)perfect direct repeat, thus potentially allowing for binding of a dimer. Our data do not allow us to clearly distinguish between monomer and dimer binding at a single P1BS site, but provides clear evidence for the interaction of proteins bound at adjacent sites. Dimerization-generated protein co-operativity has been shown to be of great importance for *in vivo* DNA-binding functions [38]; therefore, the arrangement of P1BS motifs in the promoter region might play an important role in regulating the intensity and duration of the response under Pi-limited conditions. AtPHR1 is also under regulation by SUMOylation by SIZ1 [19], and it is interesting that one of the two target lysines is within the DNA-binding domain (Figure 4), and the other is just downstream from the coiled-coil domain (Supplementary Figure S1). Thus it is quite plausible that SUMOylation of PHR1 by SIZ1 could affect DNA binding either directly and/or by influencing PHR1 interaction with itself or other proteins.

Since AtPHR1 itself is not transcriptionally up-regulated on Pi starvation and preserves a nuclear localization independently of Pi availability [16], an inhibitor of AtPHR1 or a transcription co-repressor is needed to turn off the expression of PSI genes under Pi-sufficient conditions. Recent study in *Arabidopsis* has shown that the SPX exclusive proteins AtSPX1/AtSPX2 can interact with AtPHR1 protein in the presence of Pi, resulting in reduced *in vitro* AtPHR1 binding to P1BS under increasing Pi concentrations [20]. Conversely, P1BS could only compete with AtSPX1 for AtPHR1 in the absence of Pi [20]. Similar results were reported for OsPHR2 and OsSPX1, OsSPX2 and OsSPX4 [21,22], pointing to an inhibition effect of SPX proteins on PHR1 transcription factors. This may partially explain the altered systemic and local responses to Pi in the *AtSPX1*/*AtSPX2* double mutant [20]. As only a relatively small proportion of direct PHR1-targeted PSI genes are affected in this double mutant [39], this suggests the existence of AtPHR1-independent pathways regulated by AtSPX1/AtSPX2. So far, it has only been shown in rice that OsSPX4 could partially inhibit OsPHR2 by retaining it in the cytosol [22], but an inhibition mechanism has not been demonstrated in the nucleus. In *Arabidopsis*, it was stated that ‘EMSAs showed that, in the presence of Pi, GST-SPX effectively displaced the ΔPHR1/P1BS interaction’ [20]. However, the mechanism of inhibition of PHR1 binding to P1BS was not demonstrated, and our quantitative data show that, mechanistically speaking, GST–AtSPX1 does not ‘displace the ΔPHR1/P1BS interaction’. Our SPR experiments show that AtSPX1 inhibits DNA binding by MBP–AtdPHR1, rather than removing the transcription factor that is already bound to DNA.

Although the importance of these SPX domain-containing proteins has been well recognized in plant Pi regulation [10,18,23,40], it was not until very recently that structural information has become available, possibly due to the poor stability of proteins containing this domain [35]. A study on a rice homolog demonstrated the quick turnover of OsSPX4 by the proteasome pathway, although the protein stability can be enhanced by a high level of Pi or phosphite [22]. Similar instability and protein degradation have been observed in the expression and purification of AtSPX1 in this study, and many different constructs were tested. Nevertheless, using free GST protein as a negative control, we have shown that the Pi-dependent interaction with AtPHR1 is specific to AtSPX1, indicating the recombinant AtSPX1 protein obtained is correctly folded and biologically active. In spite of the instability of many SPX proteins, structures of the SPX domain of ScVtc4 (residues 1–178), CtGde1 (residues 1–184) and HsXPR1 (residues 1–207) have been achieved [35]. Using the SPX domains from ScVtc2 and HsXPR1, NMR titrations showed a *K*_d_ for Pi between 5 and 20 mM [35]. We carried out microscale thermophoresis on GST–AtSPX1 and obtained a similar *K*_d_ for Pi between 5 and 10 mM (data not shown). However, due to some Pi binding to free GST protein, the precise affinity of AtSPX1 for Pi could not be determined. Nevertheless, when 5 mM Pi was used in the GST–AtSPX1–MBP–AtdPHR1 interaction, it was sufficient for GST–AtSPX1 to decrease MBP–AtdPHR1–DNA binding, showing the conservation of this protein domain in Pi binding capacity. On the other hand, while a *K*_d_ of ∼50 µM was reported for InsP6 binding from the OsSPX4/OsPHR2 complex [35], 100 µM InsP6 did not promote the interaction between GST–AtSPX1 and MBP–AtdPHR1. The MBP–AtdPHR1–DNA interaction was only inhibited by GST–AtSPX1 with 500 µM InsP6, suggesting that while SPX domains generally show a higher affinity to InsP6 than Pi, the InsP6-binding ability of the SPX domain could vary among closely related homologs. However, given that the InsP6 used for this experiment contains other inositol phosphate components (InsP5, InsP4 and InsP3; Supplementary Figure S3) that have reduced affinity for the SPX domain compared with InsP6 [35], the concentration of active InsP6 could be overestimated.

**Figure 5.**
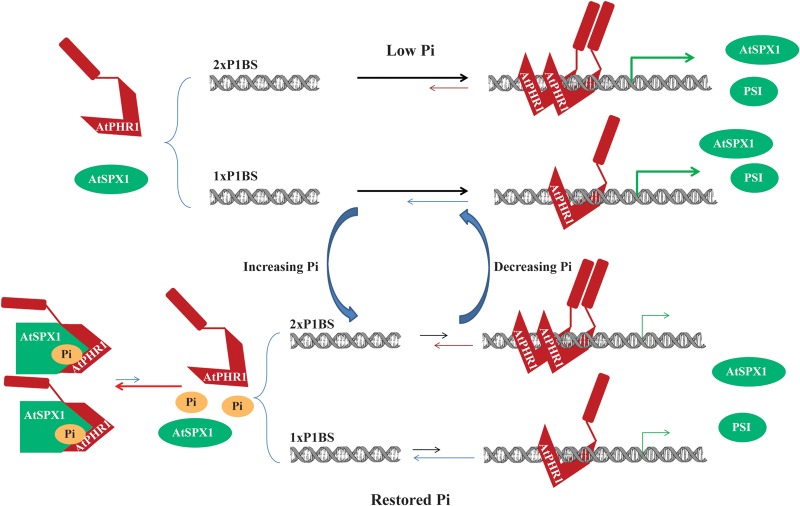
Model of how AtSPX1 might regulate PSI gene expression by influencing the AtPHR1–DNA-binding equilibrium. Under low Pi conditions, monomeric AtPHR1 in solution does not interact with AtSPX1 and the equilibrium is in favor of AtPHR1–DNA binding, leading to transcription of PSI genes including AtSPX1. Dissociation of AtPHR1 from DNA occurs over many minutes in the absence of Pi (or IP6), and interaction with 2× P1BS motifs has a slower dissociation rate compared with 1× P1BS motifs. This turnover is important for making AtPHR1 available for AtSPX1 binding and therefore receiving the signal about Pi status. Under restored Pi conditions, AtSPX1 binds to Pi (or IP6) and undergoes conformational change, and is therefore able to interact with free monomeric AtPHR1 in solution. This interaction decreases the binding of AtPHR1 to DNA by sequestering AtPHR1without changing the AtPHR1–DNA dissociation rate; therefore, it deactivates the transcription of PSI genes. Genes with multiple P1BS motifs might be turned off more slowly due to a lower dissociation rate of AtPHR1. (Other components that are also involved in the expression regulation of PSI genes are not included in this sketch diagram.)

Since our SPR data show that GST–AtSPX1 cannot disrupt the MBP–AtdPHR1–DNA interaction but was able to form a protein complex with free MBP–AtdPHR1 in the presence of Pi/InsP6 and prevent MBP–AtdPHR1 from interacting with DNA, we propose that AtSPX1 could modulate the transcriptional regulation of PSI genes by tuning the dynamic equilibrium of the AtPHR1–P1BS interaction (Figure 5). It is significant that as AtSPX1 is itself a downstream target of AtPHR1, the inhibition effect of AtSPX1 on the AtPHR1–DNA interaction could also regulate its own expression and therefore generate a negative feedback loop. Since we have shown that AtPHR1 binds co-operatively to DNA molecules, the fact that AtSPX1 cannot remove MBP–AtdPHR1 from DNA indicates that the AtSPX1-binding site on AtPHR1 is either occupied or buried in the AtPHR1–DNA complex. It would be of great interest to map the interaction domain between AtPHR1 and AtSPX1 to find out whether the interaction with AtSPX1 interferes with multimerization of AtPHR1 and whether this affects the DNA binding, or AtSPX1 binds directly to the DNA recognition helix of the Myb domain. The recently available structural information also suggests that the SPX domain undergoes a ligand binding-induced conformational change [35]. As our SPR data show that the inhibition effect of AtSPX1 on MBPdPHR1–DNA interaction is strictly Pi-dependent, this conformational change might be of great importance in the AtSPX1–AtPHR1 interaction. The same study also showed that Pi binding of yeast ScVtc2 is affected by mutations in the phosphate-binding cluster (PBC), while InsP6 binding is controlled by both the PBC and lysine surface cluster (KSC) residues [35]. Since mutations of AtPHO1 PBC and KSC resulted in reduced plant growth and enhanced PSI gene expression [35], it would be interesting to see whether these mutations would also interfere with the AtSPX1–AtPHR1 interaction and subsequently change the regulation of PSI genes.

How plants incorporate the Pi availability signal into the regulation pathways and eventually achieve the intracellular Pi homeostasis has been a focus of plant phosphorus studies. Our SPR data have provided a model in *Arabidopsis* where the Pi-sensing protein AtSPX1 regulates the AtPHR1–DNA-binding equilibrium by binding to monomeric transcription factor AtPHR1. This model offers a mechanistic basis for the transcriptional regulation in plant Pi responses.

## Abbreviations

EMSA: electrophoresis mobility shift assay
IEX: ion exchange chromatography
InsP6: inositol hexakisphosphate
InsPs: inositol polyphosphate
KSC: lysine surface cluster
P1BS: PHR1-binding sequence
Pi: inorganic phosphate
PBC: phosphate-binding cluster
PCR: polymerase chain reaction
PSI: phosphate starvation-induced
PHR1: phosphate starvation response 1
PHT1: phosphate transporter 1
P1BS: PHR1-binding sequences
SDS–PAGE: sodium dodecyl sulfate–polyacrylamide gel electrophoresis
SEC: size-exclusion chromatography
SPR: surface plasmon resonance
SUMO: small ubiquitin-like modifier
