# Supplementary material for: AtSPX1 affects the AtPHR1–DNA-binding equilibrium by binding monomeric AtPHR1 in solution
Source: Biochem J. 2017 Oct 23;474(21):3675–87. doi: 10.1042/BCJ20170522 (PMC5651819; doi:10.1042/BCJ20170522)
Supplement: Supplementary Material [file BCJ-474-3675-s1.pdf]

Supplementary Figure 1 Sequences of AtPHR1 and MBPdPHR1

A.

```
1   M E A R P V H R S G S R D L T R T S S I P S T Q K P S P V E D S F M R S D N N S Q L M S R P L G Q T
51  Y H L L S S S N G G A V G H I C S S S S S G F A T N L H Y S T M V S H E K Q Q H Y T G S S S N N A V
101 Q T P S N N D S A W C H D S L P G G F L D F H E T N P A I Q N N C Q I E D G G I A A A F D D I Q K R
151 S D W H E W A D H L I T D D D P L M S T N W N D L L L E T N S N S D S K D Q K T L Q I P Q P Q I V Q
201 Q Q P S P S V E L R P V S T T S S N S N N G T G K A R M R W T P E L H E A F V E A V N S L G G S E R
251 A T P K G V L K I M K V E G L T I Y H V K S H L Q K Y R T A R Y R P E P S E T G S P E R K L T P L E
301 H I T S L D L K G G I G I T E A L R L Q M E V Q K Q L H E Q L E I Q R N L Q L R I E E Q G K Y L Q M
351 M F E K Q N S G L T K G T A S T S D S A A K S E Q E D K K T A D S K E V P E E E T R K C E E L E S P
401 Q P K R P K I D N
```

B.

```
1   M K I E E G K L V I W I N G D K G Y N G L A E V G K K F E K D T G I K V T V E H P D K L E E K F P Q
51  V A A T G D G P D I I F W A H D R F G G Y A Q S G L L A E I T P D K A F Q D K L Y P F T W D A V R Y
101 N G K L I A Y P I A V E A L S L I Y N K D L L P N P P K T W E E I P A L D K E L K A K G K S A L M F
151 N L Q E P Y F T W P L I A A D G G Y A F K Y E N G K Y D I K D V G V D N A G A K A G L T F L V D L I
201 K N K H M N A D T D Y S I A E A A F N K G E T A M T I N G P W A W S N I D T S K V N Y G V T V L P T
251 F K G Q P S K P F V G V L S A G I N A A S P N K E L A K E F L E N Y L L T D E G L E A V N K D K P L
301 G A V A L K S Y E E E L A K D P R I A A T M E N A Q K G E I M P N I P Q M S A F W Y A V R T A V I N
351 A A S G R T V D E A L K D A Q T N S S S N N N N N N N N N L G I E G R I S E F G S E L R P V S T T
401 S S N S N N G T G K A R M R W T P E L H E A F V E A V N S L G G S E R A T P K G V L K I M K V E G L
451 T I Y H V K S H L Q K Y R T A R Y R P E P S E T G S P E R K L T P L E H I T S L D L K G G I G I T E
501 A L R L Q M E V Q K Q L H E Q L E I Q R N L Q L R I E E Q G K Y L Q M M F E K Q N S G L T K G
```

C.

```
1   M S P I L G Y W K I K G L V Q P T R L L L E Y L E E K Y E E H L Y E R D E G D K W R N K K F E L G L
51  E F P N L P Y Y I D G D V K L T Q S M A I I R Y I A D K H N M L G G C P K E R A E I S M L E G A V L
101 D I R Y G V S R I A Y S K D F E T L K V D F L S K L P E M L K M F E D R L C H K T Y L N G D H V T H
151 P D F M L Y D A L D V V L Y M D P M C L D A F P K L V C F K K R I E A I P Q I D K Y L K S S K Y I A
201 W P L Q G W Q A T F G G G D H P P K S D L V P R G S M K F G K S L S N Q I E Q T L P E W Q D K F L S
251 Y K E L K K R L K L I G S K T A D R P V K R L R L D E F S V G I S K E E I N F I Q L L E D E L E K F
301 N N F F V E K E E E Y I I R L K E F R D R I A K A K D S M E K M I K I R K E I V D F H G E M V L L E
351 N Y S A L N Y T G L V K I L K K Y D K R T G D L M R L P F I Q K V L Q Q P F Y T T D L L F K L V K E
401 S E A M L D Q I F P A N E T E S E I I Q A E L S E H K F M E S L H M K S T I A A L R V L K E I R S G
451 S S T V S V F S L P P L Q L N G L D E T W K K I P L L E Q E A K
```

Supplementary Figure 1 Amino acid sequences of AtPHR1, MBPAtdPHR1and GST-AtSPX1

A. shows the amino acid sequence of the PHR1. Grey represents the region deleted in the MBPdPHR1 construct and replaced by the MBP sequence, blue is the DNA binding domain and red is the coiled coil domain. The two lysines indicated in yellow are the targets of sumoylation by SIZ1. B. Shows the amino acid sequence of the MBPdAtPHR1 construct. MBP is shaded pale grey the other features are shaded in the same colours as A. C Sequence of GST-SPX orange is the GST, yellow is the linker and purple is the SPX1 sequence.

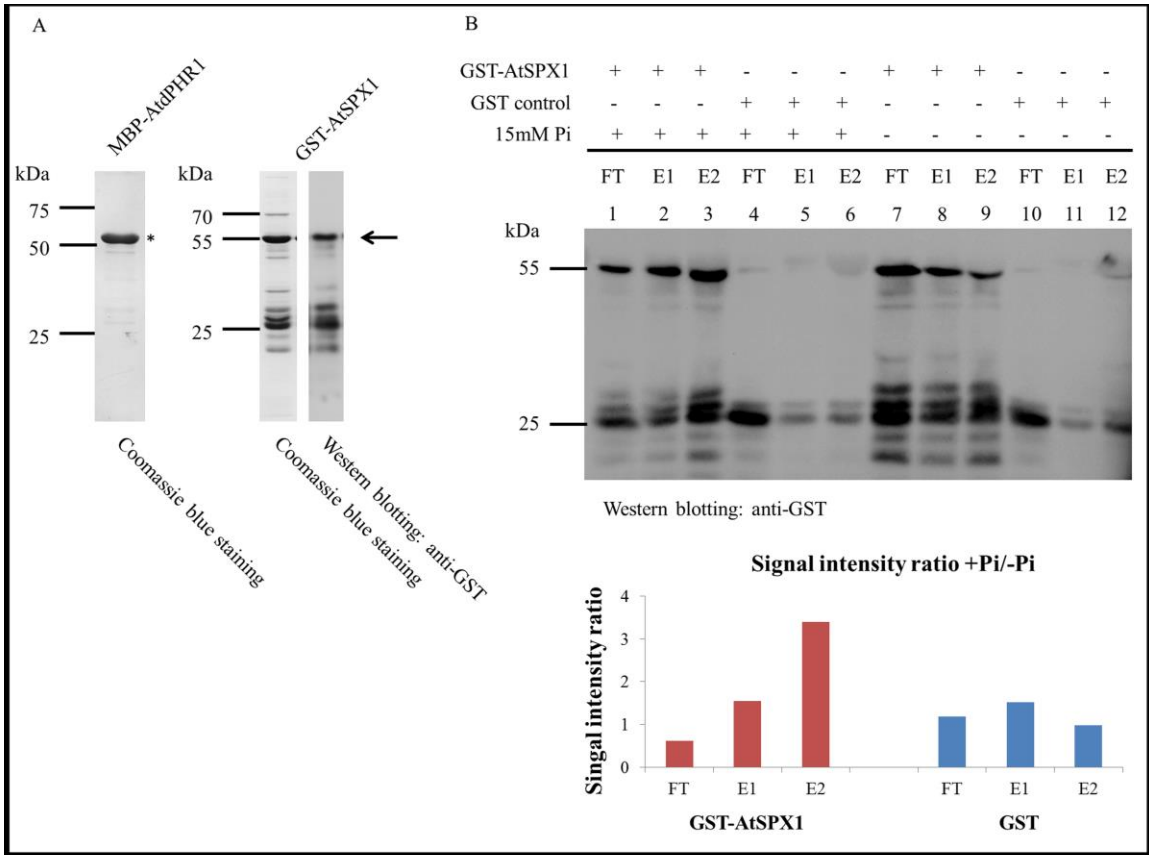

Supplementary figure 2. Affinity chromatography purification and pull-down assay of MBP-AtdPHR1 and GST-AtSPX1.

A. Elution fractions from affinity chromatography of recombinant MBP-AtdPHR1 and GST-AtSPX1whose bands are indicated by * and ←, respectively. B. MBP-AtdPHR1 and GST-AtSPX1 interact in the presence of 15 mM Pi. MBP-AtdPHR1 was immobilized on an amylose column and GST-AtSPX1 and free GST proteins were applied and incubated with and without 15 mM Pi. The presence of GST-AtSPX1 and/or free GST in the flow-through (FT) and elution fractions (E1, E2) were detected using anti-GST antibody. Quantification of this blot (B lower panel) shows that 1.5 and 3.5 fold more GST-AtSPX1 is present in fractions E1 and E2, respectively, in the presence of phosphate compared to the absence of phosphate.

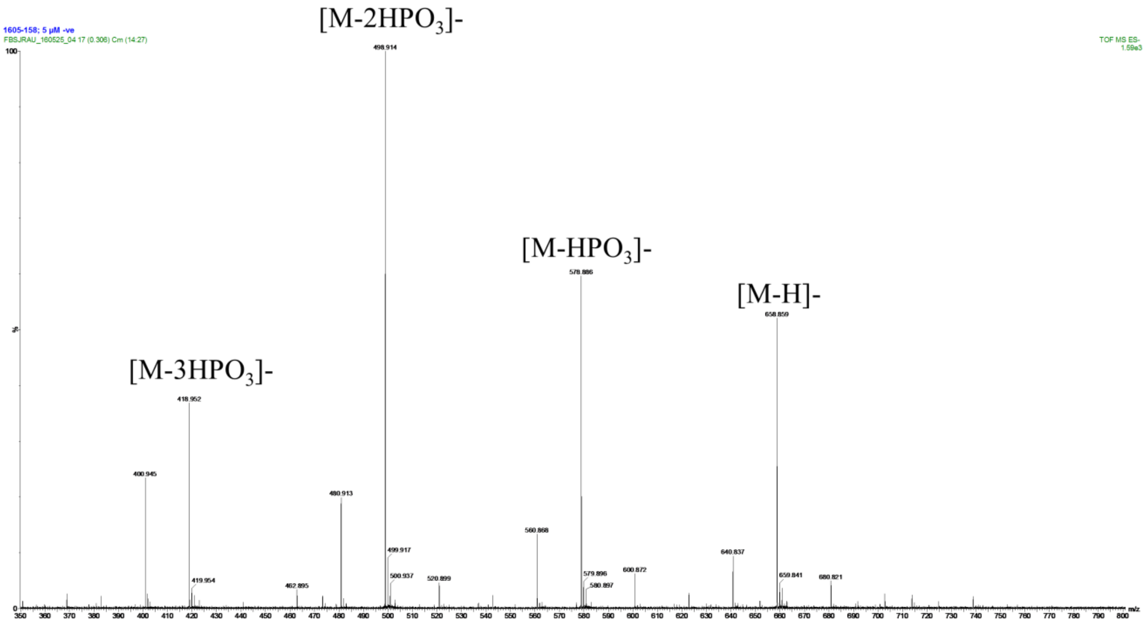

Supplementary Figure 3. Mass spectrometry analysis of inositol phosphate (InsP6) used in this experiment.

Inositol phosphate (InsP6) used in this work (Sigma P8810) was analyzed in negative ion nanoelectrospray mode. Signal at 658.859 ([M-H]-) indicates the intact, deprotonated molecule of InsP6. Other peaks observed in the spectrum correspond to loss of the phosphate (HPO_3) groups (InsP5: [M-HPO_3]-; InsP4: [M-2HPO_3]-; InsP3: [M-3HPO_3]-).
